# Understanding Asthma and Allergies by the Lens of Biodiversity and Epigenetic Changes

**DOI:** 10.3389/fimmu.2021.623737

**Published:** 2021-03-01

**Authors:** Bianca Sampaio Dotto Fiuza, Héllen Freitas Fonseca, Pedro Milet Meirelles, Cintia Rodrigues Marques, Thiago Magalhães da Silva, Camila Alexandrina Figueiredo

**Affiliations:** ^1^Instituto de Ciências da Saúde, Universidade Federal da Bahia, Salvador, Brazil; ^2^Instituto de Biologia, Universidade Federal da Bahia, Salvador, Brazil; ^3^Instituto Nacional de Ciência e Tecnologia em Estudos Interdisciplinares e Transdisciplinares em Ecologia e Evolução (IN-TREE), Salvador, Brazil; ^4^Instituto Multidisciplinar em Saúde, Universidade Federal da Bahia, Vitória da Conquista, Brazil; ^5^Departamento de Ciências Biológicas, Universidade Estadual do Sudoeste da Bahia, Jequié, Brazil

**Keywords:** asthma, allergies, holobiont, microbiome, epigenetics

## Abstract

Exposure to different organisms (bacteria, mold, virus, protozoan, helminths, among others) can induce epigenetic changes affecting the modulation of immune responses and consequently increasing the susceptibility to inflammatory diseases. Epigenomic regulatory features are highly affected during embryonic development and are responsible for the expression or repression of different genes associated with cell development and targeting/conducting immune responses. The well-known, “window of opportunity” that includes maternal and post-natal environmental exposures, which include maternal infections, microbiota, diet, drugs, and pollutant exposures are of fundamental importance to immune modulation and these events are almost always accompanied by epigenetic changes. Recently, it has been shown that these alterations could be involved in both risk and protection of allergic diseases through mechanisms, such as DNA methylation and histone modifications, which can enhance Th2 responses and maintain memory Th2 cells or decrease Treg cells differentiation. In addition, epigenetic changes may differ according to the microbial agent involved and may even influence different asthma or allergy phenotypes. In this review, we discuss how exposure to different organisms, including bacteria, viruses, and helminths can lead to epigenetic modulations and how this correlates with allergic diseases considering different genetic backgrounds of several ancestral populations.

## Introduction

Asthma and allergy are the most common chronic inflammatory diseases, especially in children (1). The prevalence of asthma is elevated in economically developed countries in Western and Eastern Europe and higher in the United States compared to other countries (2, 3). A progressive increase in the prevalence of asthma in low-income countries has also been observed (4, 5), which makes asthma prevalent worldwide. According to the World Health Organization over 80% of asthma-related deaths occur in low- and low-middle-income countries, and difficulties in accessing treatment and management are also related to that (6). On the other hand, the prevalence of eczema, allergic rhinitis, and food allergies in childhood is distributed differently between tropical countries and temperate zones (7–14). Geographic differences in the prevalence of allergies between and within populations may reflect both exposure to common environmental factors and a host genetic background, which can either increase or decrease risk (15). In terms of genetics, large genome-wide association studies (GWAS) initiatives were unable to completely explain such high and still increasing prevalence of allergic disorders as well as their phenotypic heterogeneity (1). Among the top pathways linked to asthma in such initiatives, include those related to epithelial barrier dysfunction and reduction of immune tolerance (16). In addition, studies have found not only shared but also distinct genetic components between asthma subtypes, indicating that heterogeneity is related to individual genotype (17, 18) but still do not completely explain everything.

Thus, the knowledge about the interactions between the genetic pool and the environment is increasing with several lines of evidence explaining those trends (19–21). In this context, some hypotheses explain the links between environmental changes that occurred in recent decades with the prevalence of allergies across the globe, such as urbanization, housing condition, diet, and fewer exposures to organisms such as bacteria, virus and helminths (21, 22). In fact, there is a link between the higher incidence of allergic diseases and reduced infections/exposure to organisms in Western countries and across the globe and this has been studied for several years now and appears to reflect the economy and sanitation in each territory. Additionally, the degree of industrialization and consequent changes in the habits and lifestyle of the population imply that limited exposure to several environmental factors for reducing biodiversity may contribute to an increased risk of developing or exacerbating asthma and allergies (23). David Strachan observed in 1989 that infections transmitted in early childhood, through contact between older siblings, could restrict the development of allergies (22, 24). Urbanization and improvements in hygiene, better housing conditions, and reduced chances of cross-infection in younger members of the family are the basis for what we know as the “hygiene hypothesis” (24). The initial mechanistic explanation of the hygiene hypothesis emphasized the role of Th1 cells in regulating Th2 responses. Later, the role of regulatory T cells was emphasized in the regulation of both Th1 and Th2-induced inflammatory responses through mechanisms that include the production of regulatory cytokines (25). The mechanistic pathways of the hygiene hypothesis were described extensively in the literature, other theories amplified the initial concept such as the “old friends” hypothesis (26) and, afterwards, the biodiversity hypothesis, proposed by The Karelia Allergy Study from 1998 (27). Both theories attempt to explain the impact of modifications in human living conditions and habits on the prevalence of immune-mediated diseases (28, 29).

Studies show that early exposure to antibiotics during childhood increases the risk of developing allergic diseases (30) and also regular anthelmintic use (31). Numerous epidemiologic studies reinforce that the increase in allergic diseases, eczema, and food allergies is inversely related to parasitic infections (32–36). Soil biodiversity and climatic characteristics of a country are also determinants in the types of environmental exposures and consequent development of infectious diseases and allergic sensitization. The climate and biodiversity of the tropics (fauna and flora) favor intestinal helminth infections and the dissemination of human infectious diseases transmitted by vectors like insects (37–42). According to (6), Soil-transmitted helminth infections are distributed in tropical and subtropical areas, with the highest incidence in sub-Saharan Africa, the Americas, China, and East Asia.

The tropics are also marked by sharp economic and social inequalities that reflect health and sanitary conditions and an increased risk of spreading fecal-oral transmission diseases (toxoplasmosis, giardiasis, hepatitis A, worms). In addition, the relationship between helminths and allergies is complex and is influenced by the parasite burden, chronicity, first infection or reinfection, coinfections, and parasite species present in the environment (33). In contrast, allergic sensitization to house dust mite species such as *Dermatophagoides pteronyssinus, D. farina*, and *Blomia tropicalis* is prevalent in the tropics, markedly in individuals living in better sanitary conditions and urban areas (43–45).

The importance of environmental exposures does not underestimate the fundamental participation of the family history of atopy and/or asthma and genetic background. Thus, we still have an enormous challenge to explain the occurrence of allergies and asthma. Increasing attention has been given to epigenetic modifications, i.e., modifications in DNA without sequence changes, triggered by individual exposure to environmental factors, for instance, by products of combustion, drugs, diet, and infections. Epigenetic mechanisms, such as DNA methylation and histone modifications, can modulate gene expression upon exposure to a specific environmental agent (46). Such biochemical alterations can alter different targets within the body, leading to the risk or protection of several conditions. In this review, we present the concept of holobiont and discuss how exposure to different organisms, including bacteria, viruses, and helminths, can lead to epigenetic modulations and how this modulation correlates with allergic diseases, taking into account different genetic backgrounds of several ancestral populations.

## The Context of Microbial Exposure, the Concept of Holobiont, and the Mechanisms Involved In Immune Modulation

### Holobiont Concept

Microbes are the most ancient, abundant and arguably the greatest successful form of life on Earth, contributing to the evolution and function of all more complex multicellular organisms (47). Since the early days of life, microbes interacted and established intrinsic symbiotic relationships which could evolve as a unit. The term holobiont was first coined by Lynn Margulis (48) and consisted of a simple and elegant way to explain how a host and its symbiont would evolve (49). This concept has been expanded, and it is well-accepted that a holobiont consists of a set comprised of the host and its associated microbial communities, i.e., the microbiota composed of the three domains of life, and viruses (50). According to this concept, the host (i.e., plant or animal) is subject to ecological and evolutionary pressures, so the entire community would evolve according to natural selection (51–53). This concept has been widely adopted, especially in the coral and human microbiome literature (54, 55), and it is relevant to understand its implications on human health. Understanding the relationships and interactions between microorganisms and parasites, such as helminths and protozoans, with host cells and tissues within a holistic approach is of paramount importance (49) and may provide practical solutions for challenging problems such as antibiotic resistance, allergies and asthma (56). This concept is tightly linked with the One Health framework, which is a multidisciplinary collaborative effort to achieve most appropriate health for people, animals and environment (50, 57).

The advances in DNA sequencing technologies and computational tools enabled us to explore in great detail the microbial communities and their ecological relationships on several times and space scales (58). This is a flourishing time for microbiome studies and a robust body of literature has already elucidated how environmental drivers shape free-living and host-associated microbial communities (58). Several lines of evidence show that human health is tightly linked with the equilibrium of the commensal microbial community, ultimately holobiont homeostasis. The microbial biodiversity and the relationships and interactions among microbes lead to functional outcomes. Reducing diversity, usually by a dominant microorganism, promotes a more variable and less resilient microbiota, a phenomenon known as dysbiosis, which can alter the ecosystem services provided by the microbiota, leading to a disease state.

More specifically, for the scope of the present review, the mammalian gastrointestinal tract harbors a wide diversity of microorganisms. It is estimated that *Homo sapiens* DNA makes up only a small percentage of the overall DNA on and within the human body—far greater genetic contributions are derived from bacteria, fungi, viruses, archaea, and other microorganisms as part of a vast (and individually distinct) residential community collectively known as the human microbiome (48). Additionally, more than 100 trillion microorganisms, colonize the oral–gastrointestinal tract (59). The microbiota interacts and stimulates the host immune system by activating bacterial metabolism through biochemical pathways (60), mediated by diet, host and microbiota metabolites, and antimicrobial compounds (60). The commensal microbiota is essential not only for the use of nutrients through good digestion and resistance to infections by pathogens but also supports the regulation of the host immune system, influencing innate, and adaptive immune responses (61). Dysbiosis can lead to a disruption on immune homeostasis and, consequently, to diseases such as allergy, asthma, neurodegenerative disorders, autoimmune, cardiovascular, and metabolic diseases (60, 62).

### Host-Bacterial Interactions

The presence of organisms/microbes in the human body is important to induce a proper immune response, including a regulatory mechanism that could even have a bystander effect of inflammatory conditions (63). The immune system is regulated by immune organs and cells, soluble cytokines, and cell receptors (64). The gut-associated lymphoid tissue is composed of three different lymphoid structures of the mucosa: immune cells present in the compartments of the intestinal epithelium, lamina propria, and Peyer's patches of the small intestine (61, 64). Commensal human host bacteria modulate the immune system through a bridge between epithelial cells and lymphoid structures (65). It has been previously described that microbiota can induce both Th17 and T regulatory (Treg) immune responses (66). The interaction with epithelial cells induces Th17 cell polarization and a positive regulation of antimicrobial proteins. Th17 cells are vital for protective host immunity and have been implicated in autoimmune disease development by producing the pro-inflammatory cytokines IL-17A, IL-17F, and IL-22 (59, 66).

*Clostridia*, segmented filamentous bacteria, *Bacteroides fragilis*, and other microorganisms can induce the development and/or activation of Treg cells by stimulating intestinal epithelial cells, lamina propria dendritic cells (DCs) and macrophages (59). However, it is unclear which molecular mechanisms commensal microbiota induce Treg cells in the gut (67, 68). Treg cells control autoimmune reactivity, suppress inflammatory responses, and maintain homeostasis of the microbiota (69). According to Kamada et al. (59), the reduction of Treg cells can increase the expansion of CD4+ Th cells expressing commensal bacteria-specific T cell receptors (TCRs), leading to intestinal inflammation.

In fact, the mechanisms whereby commensal microbiota can modulate immune response is an area of increasing interest. In this context, the immune cells in the Peyer's patches are responsible for the surveillance of the intestinal lumen (70). Peyer's patches contribute to the generation of B cells, which, once activated, produce intestinal secretory IgA (sIgA) (64). IgA is the most abundant class of immunoglobulin produced in mucosal tissues, mostly the gut (59, 71). sIgA is essential for the neutralization of toxins and response to pathogens. It promotes intestinal barrier function and supports maintaining host–commensal mutualism. In addition, IgA is involved in determining the diversity and regulating the composition and function of the gut microbiota (59, 70). Innate lymphoid cells (ILCs), categorized into three subsets (groups 1, 2, and 3), help also with the homeostasis, control the composition of the microbiota, contribute to the resistance to pathogens and heal the gut (59, 64). ILC1s promote homeostasis through the production of IFN-γ, while ILC2s are activated by IL-25 (induced by commensal microbiota) to release amphiregulin (Areg), which is responsible for tissue repair, and IL-5/6, which has a role in the production of IgA by B cells (72). IL-22 induces the production of ILC3s, leading to mucus production, the release of the antimicrobial peptide, fucosylation (a type of glycosylation) of the proteins from the lumina and lipids that offer energy for the microbiota (72).

Some commensal bacteria, such as *Clostridia* strains, have been shown to suppress the immune response by promoting the differentiation of Tregs and IL-10 production in the gut (65, 73). The induction of colonic Tregs can depend on *Clostridium* cluster IV and XIV and the production of metabolites, such as short-chain fatty acids (SCFAs), which have immune and metabolic functions involved in the regulation of cellular processes (74, 75). SCFAs are metabolites synthesized by bacterial fermentation of indigestible carbohydrates, in the colon, and decomposition of dietary fibers (61, 76). Propionate, butyrate, and acetate are the most predominant SCFAs in the gut and enable Treg production (73, 75). Butyrate is involved in Treg differentiation by binding G-protein-coupled receptor 43 (GPR43), a receptor of SCFAs present in colonic T cells (76). Butyrate has also been shown to induce Treg cell differentiation via dendritic cells dependent on GPR109a (77). This metabolite also can regulate central steps of the eosinophil lifecycle and function (78), inhibit ILC2 proliferation and cytokine production likely through inhibition of GATA3 expression (79), inhibit nuclear factor- κB (NF-κB) signaling via protein acetylation by a HDAC inhibitor (80) and limit the production of TNF by lipopolysaccharide (LPS)-stimulated neutrophils (81) and peripheral blood mononuclear cells (66) (Table 1).

**Table 1:** Summary of the main products (molecules) from holobionts with immunomodulatory potential and biological activities in the host.

**Microbial**	**Molecules**	**Biological activities**	**References**
*Clostridium*	SCFAs	•Anti-inflammatory activities •Regulate Treg production •Inhibit nuclear factor- κB (NF-κB) signaling •Limit the production of TNF in neutrophils and peripheral blood mononuclear cells	(60, 77, 80, 81)
*Escherichia coli*	Indoles	•Immunomodulatory function •Integrity of the enteral mucosa •Promotes epithelial cell barrier function	(62)
*Bacteroides fragilis*	Polysaccharide A	•Influences T cells fate through its •Interaction with the toll-like receptor 2.	(66)
*Candida albicans*	Candidalysin	•Induces proinflammatory cytokines, chemokines, and antimicrobial peptides	(82)
*Aspergillus fumigatus*	Gliotoxin and Fumigaclavine C	•Suppresses interferon (IFN)-γ •Downregulates Th1 cytokines •apoptosis	(83)
Eukaryotic Virus		Alteration in hematopoiese or immune activation	(84)
Bacteriophages		Production of inflammatory cytokines and type I interferon	(84)
*Schistosoma mansoni*	• Schistosomal-Derived Lysophosphatidylcholine; The soluble extract of eggs (SEA) and lacto-N-fucopentose III; Schistosomula tegument (Smteg) Sm22·6, PIII, and Sm29 antigens • Schistosomula tegument (Smteg) • Sm22·6, PIII, and Sm29 antigens	TLR2 activation •IL-10 producing Treg cells •Eosinophil recruitment •DC2 maturation •Polarization of the Th2 response. •Phosphorylation of ERK •Up-regulation of CD40 and CD86 expression •IL-12 and TNF-α production •Reduction of eosinophils in the BAL •Reduction of specific IgE •Increase in IL-10 (Sm22·6) •Reduction in IL-4 and IL-5 levels in the BAL. (PIII and Sm29)	(85–90)
*Ascaris lumbricoides*	Phosphatidylserine containing preparations (PS)	•TLR2 activation •Polarization of the Th2 response.	(91)
*Schistosoma ssp. A. lumbricoides*	Glutathione transferases	•Stimulate specific IgE antibodies	(43, 92)
*Leishmania* spp*., Toxoplasma gondii*	The glycosylphosphatidylinositol (GPI) anchors	•TLR2 and TLR4 activation induce of TNF-α	(93)

*Escherichia coli* trytophanase produces indole from tryptophan (94). This metabolite activate aryl hydrocarbon receptor, a transcription factor that induces expression of genes such as CYP4501A1, which cleans chemicals and toxins (95). Indole has an immunomodulatory function by maintaining the integrity of the enteral mucosa and promoting the epithelial barrier defense against pathogens by stimulating the production of anti-microbial peptides, mucins, and proliferation of intestinal goblet cells (62) (Table 1).

Polysaccharide produced by *Bacteroides fragilis*, a species of gut microbiota, was described to conduct systemic immunological maturity and could restore the balance between Th1 and Th2 cells and CD4+ T cell deficiency in germ-free mice (65, 66, 74). *B. fragilis* triggers toll-like receptors to create a symbiosis between the host and microbiota and affects the differentiation and development of T cells (74). *Lactobacillus reuteri* is a Gram-positive facultative anaerobic bacterium that also resides in the gut microbiota. This microorganism has many benefits as a probiotic, such as reducing infection, influencing the integrity of gut mucosa, and modulating the host's immune responses (96). *L. reuteri* has a role in protecting lung infections, stimulating the production of gut granulocyte-macrophage colony-stimulating factor, which promotes clearance of pathogens by alveolar macrophages (74, 96).

### Host-Fungus and Viruses Interactions

Although bacteria are a main component of the human microbiota, there are other organisms also composing the holobiont such as fungi, viruses, and multicellular parasites that are also important for a good balance, with potential effects on human health. The most-reported fungi in the intestines of mice and humans include *Saccharomycetes* (*Candida* and *Saccharomyces* spp.), *Eurotiomycetes* (*Aspergillus* and *Penicillium* spp.), *Tremellomycetes* (*Cryptococcus* and *Trichosporon* spp.) along with *Cladosporium, Wallemia*, and *Malassezia* spp. (97).

*Candida albicans* interacts with intestinal epithelial cells through some events, including adhesion, invasion, damage, and apoptosis (98). This interaction can lead to superficial overgrowth and epithelial invasion, followed by disease and immune activation (82). The Candidalysin, a cytolytic peptide toxin released by *C. albicans*, induces proinflammatory cytokines, chemokines and antimicrobial peptides of epithelial cells that are necessary for the recruitment of immune cells, via MAPK signaling, specifically the p38 pathway, resulting in the activation of the AP-1 transcription factor c-Fos, and the ERK1/2 pathway, leading to the activation of MKP1 (MAPK phosphatase 1), which regulates the immune response (82).

*Aspergillus fumigatus* produces a variety of precursors of toxins such as gliotoxin, which represses IFN-γ responses and induces neutrophil apoptosis through inhibition of NF-κB, a transcriptional regulator of the host proinflammatory response (99); and fumigaclavine C that down-regulates Th1 cytokines, by binding to IFN-ɤ receptor 1 (IFN-ɤ R1) (100) and induces host cell apoptosis via caspases-3,−8, and−9 (83, 101).

In addition to bacteria and fungi, the intestinal virome is composed of DNA and RNA viruses and includes eukaryotic viruses, endogenous retroviruses and bacterial viruses (102). According to (84), eukaryotic viruses and bacteriophages can stimulate changes in the immune response. Eukaryotic virus by altering the hematopoiesis or immune activation, improving a secondary infection. Bacteriophages by stimulating the production of inflammatory cytokines and type I interferon. These changes in immune responses can contribute to inflammatory diseases. In this review, we will focus in unicellular and multicellular organisms leading to immune modulation.

### Host-Helminths Interactions

Moreover, the different life cycle stages of helminths and protozoa challenge host immune responses to recognize and respond to different antigens. Distinct pattern recognition receptors members participate in the recognition of these parasites and are responsible for driving the TCD4 + cells polarization. Many molecules secreted by adult intestinal worms known as “excretory/secretory antigens” (ES) can stimulate different effects on the host's immune cells. The helminth ES products activate basophils, eosinophils, mast cells, innate lymphocyte T cells 2 (ILC2) and TCD4 + cells and drive the production of innate and adaptive cytokines. Different classes of lipids extracted from schistosome eggs and adult worms have been able to stimulate the production of several inflammatory cytokines (IL-6, IL-8, IL-10, IL-12, TNF-α). Schistosomal lysophosphatidylserine through TLR2 stimulates activation of dendritic cells with subsequent development of IL-10 producing Treg cells (85) and *Ascaris lumbricoides* derived phosphatidylserine containing preparations in the presence of interaction between TLR4 and LPS induced TLR2 with activation of TH2 response (91).

Schistosomal-Derived Lysophosphatidylcholine *in vivo* was able to induce cytokine production and eosinophil recruitment potentially through TLR2 recognition (86). Lysophosphatidylcholine participates in the recruitment of eosinophils (85) IL-5 and IL-3 stimulate eosinophilia, and recruitment is mediated mainly by chemoattractant CCL11 and CCL26 (eotaxins). Activation of eosinophils results in degranulation of chemical mediators such as Matrix metalloproteinases, cysteinyl leukotrienes, major basic protein and others (103). It has been shown that patients with *Schistosoma* infection exhibit a higher concentration of CCL3, CCL5, and CCL11 in plasma compared to uninfected individuals. These chemokines favor granulocyte recruitment, granulomatous response against egg antigens (104, 105).

Antigens from *Schistosoma mansoni*, Sm22·6 (soluble protein from the tegument of *S. mansoni*), PIII (multivalent antigen from the *S. mansoni* adult worm) and Sm29 (a membrane-bound glycoprotein from the adult worm tegument) were tested in a murine model of induced airway inflammation and showed immunomodulatory ability. These antigens induced a reduction in the number of eosinophils in bronchoalveolar lavage (BAL) and lower levels of specific IgE. In addition, Sm22·6 was associated with an increase in IL-10 while PIII and Sm29 showed a reduction in IL-4 and IL-5 levels in the BAL (90).

The soluble extract of *Schistosoma mansoni* eggs and lacto-N-fucopentose III (carbohydrates group in *S. mansoni*) has been associated with DC2 maturation and induction of the Th2 response dependent on recognition by TLR4, as well as induces phosphorylation of ERK (87, 88). In addition, schistosomula tegument (Smteg) can induce up-regulation of CD40 and CD86 expression and production of proinflammatory cytokines, such as IL-12 and TNF-α, and such activation is TLR4-dependent (89).

The glutathione transferases from helminths (*Schistosoma ssp*. and *A. lumbricoides*) stimulate specific IgE antibodies (92, 106) The glycosylphosphatidylinositol anchors from protozoan (*Leishmania* spp., *Toxoplasma gondii*) is involved in the activation of cells of lymphoid and myeloid lineage, such molecules are recognized by TLR2 and TLR4 with activation of NF-κB and subsequent induction of TNF-α in murine macrophage cells (93).

The secretion of ES products from hookworms induces activation of ILC2s and tolerogenic dendritic cells, followed by increased expression of molecules associated with tolerance and reduced expression of co-stimulatory molecules with expansion of Treg cell numbers in the gut and suppresses Th17 cell, this implies a decrease in inflammation and proliferative capacity of the parasite (107, 108). Interestingly, the Howkworms' tolerance ability was demonstrated in experimental hookworm infection in patients with celiac disease, *Necator americanus* infection suppressed gluten-induced IFNγ, IL-17, and IL-23 expression and increased the expression of IL-10, TGFβ, and IL-22 in the gut (107, 109).

It has been shown that infection with geohelminths (*A. lumbricoides, Trichuris trichiura*, hookworm) induces IL-10 and a higher mRNA expression of the Foxp3, PD-1, and regulatory molecules suppressor of cytokine signaling (SOCS) (−3) (110), reinforcing the immunomodulatory capacity of geohelminths. In addition, many of the ES components have pleiotropic immunomodulatory properties.

Taken together, it is possible to see that a balanced holobiont is necessary to maintain homeostasis. Any alteration in this environment can lead to dysregulation of the immune system and metabolism. Further studies are needed to exactly describe how holobionts changes regulate the host immune system, and which changes in its composition is associated with specific diseases.

## The Relationship Between the Shifts in Holobiont Community's Composition With Asthma and Allergies

Exposures during the peri- and post-natal periods are critical for the host's immune homeostasis, reflecting immune maturation, the development of immune tolerance mechanisms, and susceptibility to disease, also known as the first “window of opportunity” (111). This exposure includes fetal environment conditioned to the individual to the mother's lifestyle, type of delivery, diet, use of antibiotics, exposure to other children and animals, and contact with parasites and environmental microbes (112). Studies have reported that exposure to specific immunostimulatory molecules (from helminths and bacteria mainly) in childhood could reduce or block allergic disease development or progression (113). In embryonic development the immunological regulation of pregnancy is complex and an increased production of Th2 cytokines is observed, along with decreased production of Th1 cytokines. In addition, TGF-β1 appears to be involved in the differentiation of the trophoblast being an important inducer of regulatory T cells (CD4 + CD25 +) and Th17 cells, this seems to be essential for avoiding fetal allorejection (114, 115). Microbial exposures in childhood determine factors in modulation and gradual replacement for T cells and cytokines other than Th2 (116).

Moreover, universal initiatives seeking to improve the population's health conditions, such as immunization in children, improved hygiene and sanitation, access to clean water, indiscriminate use of antibiotics and anti-parasitic drugs, have been implied in reducing opportunities of microorganism's exposure/infections in early childhood with decreased Th1 responses and or decreasing Treg activation and polarizing the immune response to the Th2 profile, breaking homeostasis. Changes in the exposure of antigen patterns, including proteins released from environmental particles or infections in childhood, can impact the diversity of commensal microorganisms that make up the microbiota (117).

The use of antibiotics by mothers during pregnancy is associated with a child's asthma risk, promoting an imbalance between commensal, and pathogenic bacteria (118). Changes in the colonization of the lung microbiota of neonatal mice have (119, 120) been associated with decreased aeroallergen responsiveness induced by Helios– regulatory T cells (Helios– Treg cells) activated depending on interaction with programmed death-ligand 1 early in life, widely known as a regulator of allergic responses. Imbalance in the formation of these cells implies increased susceptibility to atopy in adulthood (119). Likewise, the altered composition of the airway microbiota is often found in asthmatic patients (120, 121). This could be explained partially by differences in environment.

### Rural vs. Urban

For instance, the prevalence and severity of asthma differ between urban and rural areas. An agricultural environment has been associated as a protective factor against the development of asthma, hay fever, and atopic sensitization in children (12, 122). An explanation would be associated with concentrations of endotoxin significantly higher in rural homes than in urban centers (123). Exposure to higher levels of endotoxin and other bacterial components in early childhood can play a protective role against allergies and asthma (123). The endotoxin constitutes the membrane of gram-negative bacteria, inducing the Th1 response by stimulating cytokines such as IL-12 and IFN-ɤ (12). In addition to that, helminth infections caused by *Ascaris lumbricoides, Trichuris trichiura*, are more prevalent among children living in areas of the rural tropics in poverty and poor access to clean water and sanitation (124).

### Helminths vs. Asthma/Allergies

Helminths and allergic asthma induce similar immune responses, including elevated serum IgE, systemic eosinophilia, and cytokines such as IL-4, IL-5, IL-9, and IL-13, the hallmark of an immune Th2 response (125). Additionally, basophils, mast cells, neutrophils and innate lymphoid cells are involved (126). Interestingly, infections by parasite species such as *A. lumbricoides, Schistosoma mansoni, Strongyloides stercoralis*, and *T. trichiura*, have been associated with a reduction in airway allergic inflammation (34) with decreased Th1 responses (Table 2). The immunomodulatory ability of geohelminths to reduce susceptibility to allergies in humans has been recognized, and it is related to the immune-regulatory network, including helminth-derived products. Recombinant proteins of *S. mansoni* were associated with an increase in IL-10 and TGF-β, an increased frequency of regulatory T and B cells, and a reduction in the frequency of activated T lymphocytes that produce IL-4 and IL-13 in individuals with severe asthma and animal models (142, 143). In addition, *T. trichiura* infection appears to modulate the immune response among asthmatics, with some studies reporting a risk association for asthma among infected people and positively associated with wheezing (33, 35, 36).

**Table 2:** Summary of the immunomodulatory effects of some halobiont's organisms on asthma and allergy.

**Holobiont**	**Immunomodulatory effects**	**Consequences**
*Schistosoma mansoni*	↑TNF-a and IFN-g in acute phase ↑ IL-10 in chronic phase	Prevent against the development of allergies and asthma (32, 34) Down-modulate the inflammatory response in murine model of ovalbumin (OVA)-induced airway inflammation (90)
*Ascaris lumbricoides*.	↑IL-4, IL-5, and IL-10	*Ascaris lumbricoides* eggs was associated with an increased prevalence of asthma (124) Reduced risk of wheeze (127) Anti-A lumbricoides IgE antibodies were associated with risk of wheezing in atopic children and atopia (36)
*Trichuris trichiuria*	Modulation of pro and anti-inflammatory cytokine (35) ↓TNF-α and IL-6 levels among asthmatics infected ↑IL-10	↓allergen skin test reactivity (33) Positively associated with wheezing (36)
*Helicobacter pylori*	Th1 polarization ↓Th2 response ↑ (IFN)-γ, IL-12, IL-18, IL-23 (128)	Negative association between H. pylori infection and asthma, eczema, and rhinitis (128)
Hookworm (*Ancylostoma duodenale* and *Necator americanus*)	Induction of IL-25 and ILC2s (129) ↑IgG1, IgG4, and IgE Expansion of Treg cell numbers in the gut (107, 108)	Protect against wheezing, asthma, and allergic diseases. Reduction in risk of wheeze (130)
*Toxoplasma gondii*	Induces IL-10 production, IL-27, and activity of lipoxins (131, 132).	Protective effect against atopy (133) Suspend the development of airway inflammation and atopy in mice (133) Decrease in specific IgE for *Dermatophagoides pteronyssinus* (134)
Toxocara spp.	↑ levels of total IgE Cross reactivity with aeroallergens	Positive skin tests to allergens, and asthma prevalence and morbidity (135, 136)
*Bifidobacterium*	Stimulating IL-10 or IL-12 synthesis	Protective factor for high risk of allergic asthma and atopic dermatitis in children from Turkey (137)
*Bacteroides fragilis*	Stimulate Th2 cytokines by biding TLR2	Risk factor in children with a positive API (138)
*Penicillium*	High counts in patients with atopy	Risk factor for atopic asthma (139, 140)
*Aspergillus fumigatus*	Decrease the expression of GCR	Aggravate airway hyper-responsiveness and increase the level of TLR2 (141)

*↑increase; ↓decrease*.

Maternal soil-transmitted helminths (STH) infections can sensitize the individual still in the fetal phase. Cooper et al. (144) reported a strong association between Maternal STH infections during pregnancy (mainly moderate to chronic *A. lumbricoides* infection) and childhood STH infections. During pregnancy, infected mothers have an increased number of CD4 + T cells and production of IL-10 in cord blood from newborns demonstrating immunomodulation mediated by parasite antigens (145, 146). In the same study, poor hygiene conditions, with the prevalence of STH infections, were not associated with reduced eczema-asthma-rhinitis symptoms (144). Co-exposure to mites and *Ascaris lumbricoides* in the context of low worm burdens promotes allergic sensitization and asthmatic symptoms by increasing parasite-specific IgE production, mite-specific and mite–parasite cross-reacting IgE antibodies, observed mainly in urban areas, once in rural areas the exposure to helminths tends to be chronic (40). *A. lumbricoides* extract was associated with inhibition of pulmonary eosinophilia in mice sensitized with ovalbumin (OVA) and a decrease in allergic inflammation independent of IL-10 (147) (Table 2). In contrast, Anti-*A. lumbricoides* IgE (but not active infection), were associated with risk of wheezing in atopy in atopic children (36).

### Viruses and Protozoans vs. Asthma/Allergies

Some viral and protozoan infections have been associated with decreased reactivity to skin prick tests for aeroallergens (148, 149) and asthma (150). The host's defenses against viruses are marked by a predominance of the Th1 response and interaction with different Toll-like receptors with probable biological and immunomodulatory effects on Th2 responses. *Toxoplasma gondii* infection has been reported to suspend the development of airway inflammation and atopy in mice (133) and induces IL-10 production, IL-27 and activity of lipoxins (131, 132). In addition, a negative association was reported between *T gondii* seropositivity and specific IgE to *Dermatophagoides pteronyssinus* (134). Hepatitis A virus (HAV) exposure has been inversely associated with allergies (151). In the United States, positive serology for HAV was associated with a lower chance of developing hay fever and asthma and skin reactivity to airborne allergens (152). In Turkey, the prevalence of atopy was lower among individuals with positive serology for HAV and hepatitis B virus (anti-HAV IgG, HBsAg, anti-HBc IgG) (153). The accumulated infection burden, considering HAV, herpes simplex virus, Epstein–Barr virus, Cytomegalovirus, *Helicobacter pylori*, and *Toxoplasma gondii* (> 3 microbes), was associated with a protective effect against atopy (149, 153). According to Amedei et al. (128) *H. pylori* infection was negatively associated with asthma, eczema and rhinitis and induces Th1 polarization (Table 2). Moreover, BCG vaccination at an earlier age was associated with a decreased risk of atopy in children without a family history of asthma and atopy (154). However, there are controversies regarding some types of vaccines (155, 156).

### Bacteria vs. Asthma/Allergies

A study (157) from the Copenhagen Prospective Study on Asthma in Childhood has shown that the lack of development of the gut microbiome in the first year of life is the determinant to the occurrence of childhood asthma, increasing asthma risk. The lower number of *Lachnospiraceae* and *Ruminococcaceae* genera was observed in asthmatic children and was associated with allergic wheezy phenotype (157). The production of SCFAs was suggested to be associated with asthma development in a study of high vegetable fiber intake by children from Manitoba Prospective Cohort Study of Allergy, Genes and the Environment, acting as a protective factor against to airway hyperresponsiveness (158, 159). *Bifidobacterium longum* has been described influencing the prevalence of allergic disease being a protective factor for allergic asthma and atopic dermatitis in children from Turkey (Table 2) (137). In contrast, *Bacteroides fragilis* count was significantly higher in children with a positive Asthma Predictive Index as compared with those negative (138). It seems that *Bacteroides* species maybe stimulate Th2 cytokines and some studies have found an association between this genera and higher IgG in children with allergies (138) (Table 2).

### Fungus vs. Asthma/Allergies

Skin-test for fungal allergens is usually characterized with the presence of immediate cutaneous hyperreactivity or positive results for specific IgE antibodies to fungal antigens and has been related to be especially common in patients with life-threatening asthma (139, 160). *Aspergillus, Alternaria, Penicillium, Cladosporium*, and *Trichophyton*, have been described to be associated with exacerbation and severity of asthma (139). *Penicillium* species was higher in patients with atopy compared with healthy control subjects, suggesting to be a risk factor for atopic asthma since this genera is one of the most common fungi related to allergic asthma exacerbations among adults (Table 2) (140, 161). A study using rats with asthma shows that *Aspergillus fumigatus* may decrease the expression of glucocorticoid receptor aggravating airway hyper-responsiveness and increase the level of TLR2, involved in airway inflammation (141) (Table 2).

The immune response in the context of asthma and atopy as well as its development, differentiation of cell subtypes and expression of receptors and cytokines are influenced by exposures to holobionts. This immunological modulation is often accompanied by epigenetic changes. In part, such modifications that allow such plasticity of immune responses, also promote homeostasis through the balance of adaptive immune responses in certain conditions and are responsible for the maintenance and intensification of Th2 responses, increasing the risk for allergic diseases and other inflammatory diseases.

## Epigenetic Mechanisms: Basic Concepts

Currently, epigenetics can be defined as changes above the DNA without changing the nucleotide sequence (162). Different mechanisms of epigenetic regulation have been described, such as DNA methylation, histone modifications and non-coding RNAs. Since the first Waddington epigenetics works (163, 164), many studies have been conducted to determine the influences of epigenetics in several conditions. The epigenetic mechanisms are widespread in the different cell types of the human body, including cells that participate in an immune response pathway directly involved in the etiopathogenesis of asthma and other allergic diseases. Understanding the impact of epigenetic changes on the normal and abnormal functioning of these cells, therefore, is an important piece to compose the complex puzzle that allergic diseases represent. Bellow, are described the main mechanisms of epigenetics-induced changes in gene expression.

### DNA Methylation

DNA methylation is the addition of a methyl group (CH3) to a cytosine by DNA methyltransferases, generating 5-methyl-cytosine (165). Promoter regions of genes have a large amount of CpG (cytosine-phosphate-guanine), known as CpG islands, that when methylated prevents the binding of transcription factors and represses gene expression (166).

Several factors can contribute to DNA methylation changes, such as aging, environmental exposure, cell type, and age. These modifications can be passed through cell division through either mitosis or meiosis (167). Recently, many epigenome-wide association studies (EWAS) have described the association between DNA methylation and asthma, and several genes were identified, including EPX, IL4, IL5RA, PRG2, SIGLEC8, CLU, AP2A2, and KCNH2 (168–170).

### Histone Modifications

Histone is a protein involved in the organization of chromatin and regulation of gene expression. They are grouped into 8 subunits, two of each H2A, H2B, H3, and H4 forming an octameric nucleosome where the DNA coils. Histone H1 is associated with this complex and stabilizes the chromatin structure. Some modifications can occur in the N-terminal tails of histones, including acetylation, methylation, ubiquitylation, and phosphorylation (171).

Histone acetylation occurs when acetyltransferases add lysine residues to histone tails. Histone acetylation increases DNA access and facilitates the process of transcription, increasing gene expression. Previous studies reported that H3K4me3 and H3K27me3 were associated with T helper cell differentiation and IL-5 expression (172), and higher histone 3 acetylation levels at the IL13 locus were associated with higher protein levels of IL13 (173).

Methylation in histones is performed by methyltransferases and usually occurs at lysine (K) or arginine (A) residues and can increase or decrease gene expression depending on the modified residue. For instance, inactivation can occur by methylation on H3K9, H3K27, and H4K20, while activation occurs by methylation on H3K4 and H3K36 (174).

### Non-coding RNAs

Non-coding RNAs are a group of RNAs that do not encode proteins but can play an important role in the regulation of gene expression acting at the post-transcriptional level (175). Regarding size, RNAs with regulatory functions are divided into short non-coding RNAs (siRNAs, miRNAs and piRNAs) and long non-coding RNAs (lncRNAs) (176). They can silence genes through the RNA interference pathway and modulate several biological processes, including immunological functions (177).

## Shaping Immune Responses Through Epigenetics Mechanisms: Regulation of Cytokine Gene Expression, Transcription Factors, and Regulation of Immune Responses In Asthma and Allergy

The epigenetic mechanisms previously described are present in the different contexts and cell types of the human body, including driven immune cell pathways directly involved in the etiopathogenesis of asthma and other allergic diseases. Understanding the impact of epigenetic changes on the normal and abnormal functioning of these cells, therefore, is an important piece to compose the complex puzzle that allergic diseases represent. Some advances in this direction have recently been achieved. Thus, epigenetic modifications play a role in regulating the expression of cytokines related to T cell differentiation and transcription factors (178). The development of cell types and, consequently, the specificity of immunological responses occur through internal stimuli or driven by stimulatory molecules of microorganisms. They act on surface receptors such as TLR signaling, signal transduction proteins, and lineage-specifying transcription factors, promoting intracellular events. Even the development of T lymphocytes and maturation for helper (CD4+) and cytotoxic (CD8+) cells are influenced by epigenetic control. This promotes CD4+ silencing in CD8+ thymocytes and the development of T helper cell subsets (Th1, Th2, and Th17) accompanied by epigenetic changes (179, 180). Epigenetic changes have also been linked to the activation and polarization of macrophages (M1/M2 phenotypes) (181).

Allergic diseases, e.g., asthma, result from a strong interaction of genetic and environmental components with remarkable phenotypic heterogeneity. This heterogeneity of asthma can be partially explained by dysregulated epigenetic mechanisms correlated with environmental exposures, pharmacological treatments, and airway inflammation and function (182). There is evidence that the induction of Th2 cells, maintenance, and the resurgence of memory Th2 cells are controlled by epigenetic regulation since this induction is mediated by signal transducer and activator of transcription 6 and the consequent production of the Th2 cytokine profile (183). Hypermethylation in GATA3 CpG loci was associated with a decreased risk of asthma at birth (184), and hypomethylation of IL-13 and interleukin 5 receptor subunit alpha (IL5RA) was associated with an increased risk of asthma in teenagers (169).

Epigenetic mechanisms are essential in controlling gene expression or silencing and the consequent balance of Th1/Th2 responses. Corroborating the principle that Th1/Th2 imbalance is involved in the pathogenesis of asthma and atopy, experimental studies in mice showed hypermethylation in the IFN-ɤ gene promoter in TCD4+ cells, leading to the silencing of the *IFNG* gene (Th1 pattern) (185). During initiation of a Th2 immune response, an increase in histone acetylation was observed at the Th2 cytokine *loci*. It has been demonstrated that the *IL4* and *IL13* genes are hypomethylated in asthmatic patients, critical genes in amplifying the Th2 response (186).

A balance of histone deacetylase (HDAC) and histone acetyltransferase (HAT) activity has been considered to regulate gene expression. Reduced HDAC expression was observed among adults with severe asthma compared to mild asthma (187). In atopic asthmatic children, a relationship was found between HDAC/HAT activity and increased histone acetylation, and the degree of acetylation was associated with an increase in bronchial hyperresponsiveness (188).

miRNAs have also shown an essential role in the inflammatory response of asthma. Studies have shown that miR-155 and miR-221 are associated with modulation of the Th2 response (189) and hyperproliferation of airway smooth muscle in asthmatic patients, respectively (190). In the asthma context, non-coding RNAs have been observed as markers of disease diagnosis, phenotypes, and response to treatments. For example, a negative correlation between the levels of miR-323-3p and IL22 and IL17 was observed in PBMCs from patients with asthma, suggesting that non-coding RNA acts as negative feedback in the production of these cytokines influencing the immune response of these individuals (191). Moreover, elevated levels of miRNA-21 in the peripheral blood of children with asthma were identified, suggesting that this non-coding RNA may be a biomarker in the diagnosis of asthma (192). Furthermore, high expression of microRNA-155 and decreased expression of Let-7a were observed in the plasma of asthmatic patients and were associated with the degree of asthma severity, suggesting that these markers can be used both in diagnosis and in the prediction of the severity of disease (193).

In the context of regulatory T cells (Tregs), cells that play a substantial role in immune homeostasis through mechanisms of tolerance and immune de-activation during a regular immune response and suppression of a self-destructive immune response, the repressive phenotype of Tregs is conferred, in part, by the expression of Forkhead box protein 3 (FOXP3) (194). Hypermethylation of CpG islands in the promoter region in the *FOXP3* locus impacts transcriptional silencing and consequent reduction in Treg cell function. Air pollutants have been recognized as acting on epigenetic changes. Increased exposure to polycyclic aromatic hydrocarbons has been associated with an increase in DNA methylation at the *FOXP3* locus in peripheral blood mononuclear cells and elevated total IgE with significant effects on asthmatics (195). Furthermore, an increase in *FOXP3* DNA methylation has been associated with an increased risk of asthma and persistent wheezing (196).

**Figure 1:**
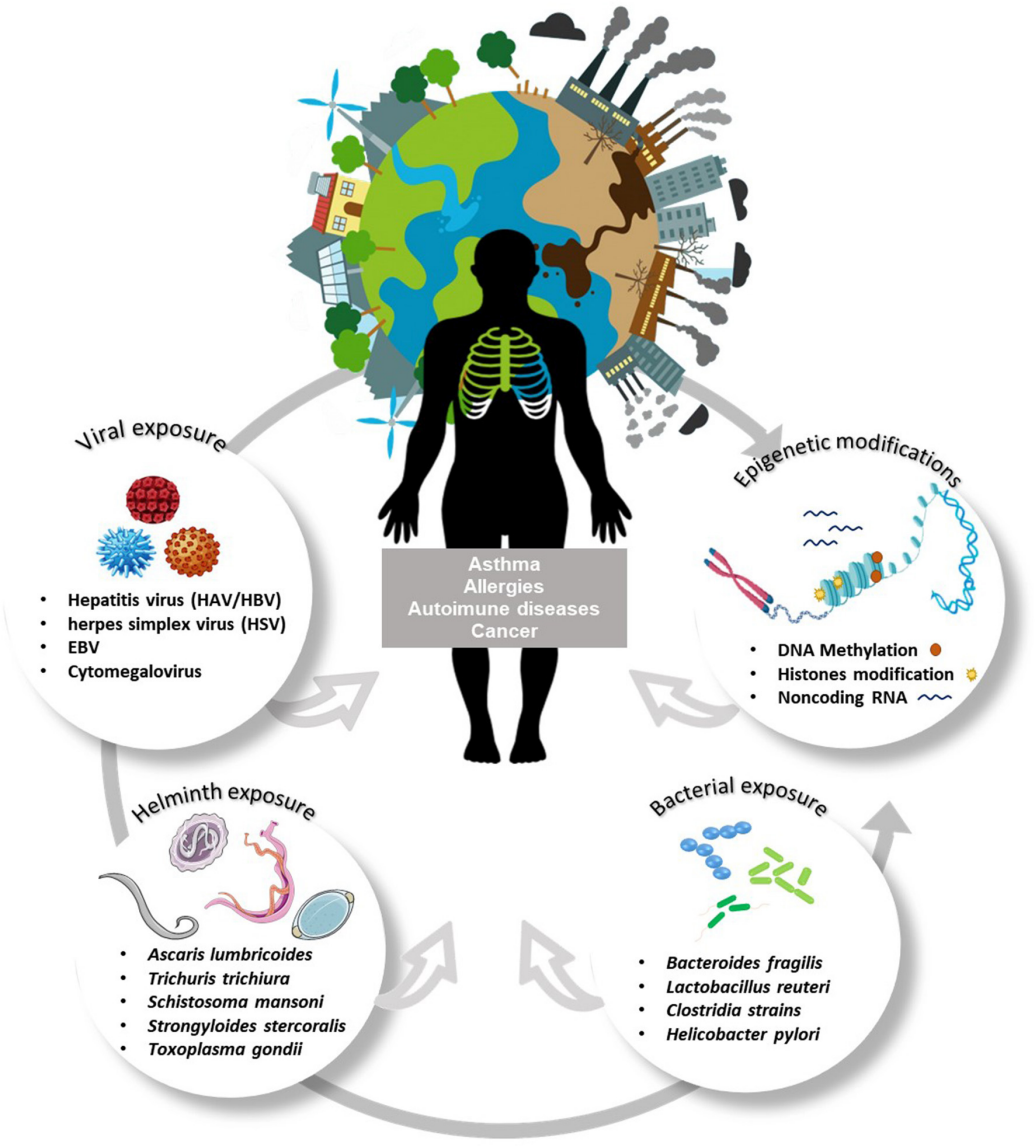
Interaction between multiple environmental exposures and epigenetic changes: impact on immune-mediated diseases. Polluting agents, environmental exposure, diet, age, drugs, and especially exposure to organisms (species of bacteria, fungi, protozoan, and helminths) act as inducers of epigenetic changes. Among the epigenetic modifications are DNA methylation and histone modifications. Histone acetylation increases DNA access and facilitates the process of transcription, increasing gene expression. The addition of a methyl group in CpG islands prevents the binding of transcription factors and represses gene expression. The interaction between environmental exposures and epigenetic variations begins in the embryonic period and continues throughout life, being strongly dependent on the environmental experiences/challenges of everyone. Such alterations can be highly modifiable by instantaneous adaptation to the environment or generate inheritable epigenetic patterns and consequences to offspring. Epigenetic mechanisms influence cell differentiation and polarization of immune responses, these events modulate biological responses, and can interfere with the development of different immune-mediated diseases such as cancer, asthma, allergies, autoimmune diseases.

Figure 1 shows different factors shaping asthma and allergy, such as environmental factors, epigenetic changes, and exposure to holobionts components which can be modulated by disturbances in the homeostasis.

## Epigenetic Changes Associated With Holobiont Interactions

Since immune dysregulation is linked to allergies and asthma through the lack of certain environmental exposure, one could think that potential epigenetic mechanisms may play a role in this phenomenon. A question raised by these new findings is at “what point in the development of the human being the epigenetic mechanisms could act to drive the maturation of the immune system in early life?.” In this sense, in recent years, the hygiene hypothesis has been expanded to encompass the potential effect of prenatal exposure to microbial agents on modulating the individual risk of asthma and other allergic diseases (115). Although some evidence in this regard was already available through epidemiological studies that assessed the impact of maternal microbial exposure on the risk of developing allergic conditions in the offspring (116), the elucidation of the molecular mechanisms underlying these processes has been a relatively new and fascinating field of investigation.

Alterations in the gut microbiota, called dysbiosis, is related to infections and inflammatory diseases and comes with irregular immune responses, e.g., particular inflammatory cytokines (66, 75). Changed gut microbiota can also increase the production of NK-κ-B and TNF-α and the overexpression and activation of Th1 and Th17 cells (197). Studies have shown that changes in the gut bacterial composition and the production of its metabolites can influence epigenetic levels, such as reducing methylation and inhibiting histone deacetylases (197). Specifically, the metabolites influencing epigenetic enzyme activity are a substrate needed for epigenetic changes (197). For example, Butyrate, a metabolite from microbiota, can also inhibit HDAC, increasing the expression of FOXP3 through the acetylation of histone H3 in the promoter and enhancing Treg generation (164).

One of the first mechanistic studies on the allergoprotective effects of maternal exposure to microbes used a mouse model with the farm bacterium *Acinetobacter lwoffii* (198). In this study, the protective effect for allergic airway inflammation (AAI) in the offspring was dependent on maternal TLR signaling, since this protection was abolished when mothers were knocked out for multiple TLR genes. The authors also demonstrated that the immune dampening observed in the progeny of pregnant mice was not due to microbial components able to pass the fetus-maternal interface and directly activate the developing fetal immune system. This last observation suggests the possible involvement of epigenetic factors operating in the fetuses of mothers exposed to *Acinetobacter lwoffii*. Indeed, another study by the same group reported epigenetic changes in Th1/Th2 cytokine genes in offspring from pregnant mice exposed to *A. lwoffii* (199). While the IFNγ promoter on CD4+ T cells exhibited significant protection against the loss of histone 4 (H4) acetylation, with the consequent increase in IFN-ɤ expression in OVA-induced AAI, the IL4 promoter showed a significant decrease in H4 acetylation and diminished gene expression. A protective effect against induced AAI has also been shown in the progeny of pregnant mice exposed to *Helicobacter pylori* extracts (200). An epigenetic consequence observed in the offspring was the enhanced demethylation of the regulatory T cell-specific demethylated region in Foxp3+ Treg cells. Intriguingly, this protective effect extended to the second generation (F2) of mice exposed to *H. pylori* antigens during pregnancy, with both sexes exhibiting similar levels of protection. This indicates that the epigenetic changes in the offspring induced by transmaternal exposure to *H. pylori* may extend to chromosomal *loci* other than just the TSDR linked to the X chromosome. The transfer of allergoprotective effects during the prenatal phase through maternal infection with the helminth *Schistosoma mansoni* has also been previously investigated in experimental models of AAI in mice (201). Interestingly, this protective effect was dependent on the stage of the immune responses to *S. mansoni* in the females at the time of mating. While the offspring of the mothers mated during the Th1 and regulatory phases showed protection against OVA-induced AAI, those born to mothers mated during the Th2 phase showed an exacerbation of the allergic inflammatory response compared to the controls. The authors also demonstrated that the protective effect of transmaternal exposure to *S mansoni* was mediated by maternally produced IFN-ɤ and not by the transfer of helminth antigens to the fetus. Potential epigenetic changes in the offspring associated with the protective immune phenotype, however, were not further investigated and remain to be clarified (202).

In humans, data on epigenetic changes induced by pre- or post-natal exposure to microbial agents and their relationship to asthma and other allergic conditions are still scarce. A pilot study evaluated the effect of maternal exposure to the farm environment on offspring epigenetic changes for genes known to be associated with asthma and allergies (203). Significant differences between non-asthmatic children born to mothers exposed to the farm environment and asthmatic children born to unexposed mothers were observed for the methylation pattern of the *ORMDL3* and *STAT6* genes in cord blood. In a recent study, Lund et al. (204) reported that changes in the methylation pattern in chromosomal previously linked to asthma, such as the SMAD3 promoter at 15q22.33 and intronic regions of the DDO/METTL24 genes at 6q21, were associated with atopic asthma in children with early rhinovirus-induced wheezing. In turn, DNA methylation changes linked to the prostaglandin D2 synthase gene were associated with non-atopic asthma in children with rhinovirus etiology at the first severe wheezing episode (204). This suggests that the epigenetic changes triggered by the same microbial agent may differ according to the specific phenotype of asthma or other allergic diseases, which needs to be further investigated in the future.

Taken together, although several studies related to epigenetics of asthma and allergies have been published so far, very few initiatives explores the role of the environmental changes, in special, exposure to organisms such as bacteria, fungi, protozoan and helminths as important modulators of those biochemical changes in human DNA. Further studies are needed to better understand such associations.

## Future Therapies as Potential Modulators of Epigenetics Changes in Asthma and Allergies: Observations and Future Perspectives

The usual immunotherapy and pharmacological therapy in the treatment of asthma and allergies act in the modulation of immune responses, with a focus on reducing inflammation and increasing immunological tolerance. This immunological modulation is almost always accompanied by epigenetic changes. It is even possible to distinguish different epigenetic signatures between untreated individuals and individuals under treatment (205).

The use of inhaled corticosteroids (ICS) in the management of moderate to severe asthma is recommended by asthma management guidelines (206) and several studies have shown that corticosteroids are potent epigenetic modifiers (207–209). Children with better response to corticosteroids have been shown to have hypermethylation in Vanin-1 (VNN1) promoter compared to the group with poor response, in addition VNN1 mRNA expression was higher among good responders. VNN1 appears to have an important role in corticosteroid responsiveness among asthmatics, and can be used as a biomarker for treatment response (209, 210). Acetylation of histones by HATs activity was reported to be reduced in asthmatics treated with inhaled steroids (211). Variations in serum IgE concentrations can be influenced by DNA methylation patterns. An association between total serum IgE concentration and low methylation at 36 *loci* has been demonstrated, this observation may be useful in optimizing therapies with anti-IgE antibodies such as omalizumab (212).

Studies evaluating the effectiveness of peanut oral immunotherapy found a great suppressive function of Treg cells and higher levels of FOXP3 hypomethylation among treated individuals (213). In addition, a study involving cow's milk allergy children and dietary intervention using probiotic *Lactobacillus rhamnosus* (abundant in butyrate-producer bacteria strains) demonstrated that oral tolerance in children with IgE-mediated CMA involves epigenetic regulation of the FOXP3 gene. Difference in the methylation status of FOXP3 was found among children who developed oral tolerance after probiotic therapy (205). Prenatal administration of *Acinetobacter lwoffii* F78 in murine demonstrated a modulation in Th1/Th2 balance genes, with protection for asthma in the progeny, accompanied by changes in DNA acetylation (199).

Although some studies using probiotic supplementation in animal models have indicated a protective effect of probiotics on asthma and allergic Rhinitis (214, 215), studies in humans are still limited due to couple limitations such as the duration of supplementation.

Many efforts have been focused on understanding and developing microbial therapies using technological approaches involving parasitology, genomics, transcriptomics, and proteomics methods. Currently with the help of bioinformatics and helminth genome sequencing initiatives it is possible through *in silico* analyzes to identify molecules with potential immunomodulatory properties. These databases are available on WormBase Parasite, HelmDB, and Heminth.net (216–218).

The identification of genomic sequences of helminth parasites known to down-modulate the immune system of mammalian hosts such as *Ascaris suum, Necator americanus, Schistosoma mansoni, Strongyloides* spp. as mentioned in previous topics in this review, have motivated the development of recombinant helminth proteins with therapeutic potential for immune-mediated diseases such as protease inhibitors, cytokine homologs and lectins (219). High immunogenicity has been observed for these therapeutic recombinant proteins (220) which may be able to mimic the immunomodulation observed in helminth infections. However, standardized studies in humans as well as adequacy of doses and treatment duration are still necessary.

Epigenetic mechanisms play an important role in the regulation of immune response and are strongly influenced by microbial exposures and drug use, advancing the knowledge about such interactions may be used to both development of future target therapeutic strategies for asthma and allergies but also to discover new biological properties in current drugs in use.

The genetic susceptibility to allergic disorders is known be polygenic and recent studies have established that the presence of the gut microbiota is essential for normal gene expression (221, 222). The presence of certain bacterial species in the gut, such as *Helicobacter pylori* increases the CpG methylation in the promoter region of O6-methylguanine DNA methyltransferase, which ends up decreasing the expression of this DNA methyltransferase in gastric mucosa cells (222).

*Lactobacilli* and *Bifidobacteria* are the major source of butyrate and the absence of these species is important. By inhibiting HDACs, butyrate suppresses nuclear NF-κB activation, upregulates PPARɤ expression, and decreases IFNɤ production in the residing gut immune cells, promoting an anti-inflammatory gut environment (222). In a study with patients with allergic rhinitis, blocking the HDAC activity restored the integrity of the nasal epithelium and restored mucosal function and prevented the development of airway inflammation and hyperresponsiveness in experimental models (223).

Studies in dietary manipulation have demonstrated that diets high in methyl-donating nutrients are associated with hypermethylation of the epigenome, impacting the gene expression, especially during early development when the epigenome is first established, and can have long-term effects in adult life (224, 225). According to Bae et al. (225), in humans, methyl donors for DNA methylation are mostly derived from dietary methyl groups nutrients such as folate, vitamin B12, and choline. Methyl donors affect DNA methylation and immune responses such as Th17, Th1/Th2 balance, and Treg generation (225).

Additional studies are needed to better characterize the mechanisms underlying the different asthma phenotypes and their correlation with clinical characteristics, and those that contemplate the complex interaction of different epigenetic mechanisms and those that focus on a single-cell type or investigations at the single cell level (221, 226). In this sense, EWAS can be useful to identify patterns of epigenetic signatures among asthma and allergy phenotypes and clinical characteristics, which reinforces the potential of epigenetic changes as future biomarkers for diagnosis and target personalized therapies.

## Author Contributions

BF, HF, PM, CM, TS, and CF have contributed for the first draft. HF designed the figure. CF designed the work. All authors listed co-authored and proofread the manuscript and approved the final manuscript.

## Conflict of Interest

The authors declare that the research was conducted in the absence of any commercial or financial relationships that could be construed as a potential conflict of interest.

## Abbreviations

AAI: allergic airway inflammation
BCG: Bacillus Calmette–Guérin
CpG: cytosine-phosphate-guanine
ERK: Extracellular signal-regulated kinases
ES: excretory/secretory antigens
EWAS: epigenome-wide association studies
GWAS: genome-wide association studies
HAT: histone acetyltransferase
HAV: Hepatitis A virus
HDAC: histone deacetylase
ICS: inhaled corticosteroids
MAPK: mitogen-activated protein kinase
miRNAs: MicroRNA
SOCS: suppressor of cytokine signaling
STH: soil-transmitted helminths
VNN1: Vanin-1.
